# Supplementary material for: Cryptotympana pustulata Extract and Its Main Active Component, Oleic Acid, Inhibit Ovalbumin-Induced Allergic Airway Inflammation through Inhibition of Th2/GATA-3 and Interleukin-17/RORγt Signaling Pathways in Asthmatic Mice
Source: Molecules. 2021 Mar 25;26(7):1854. doi: 10.3390/molecules26071854 (PMC8037444; doi:10.3390/molecules26071854)
Supplement: Supplementary file 1 [file molecules-26-01854-s001.zip › molecules-1141585-supplementary data (Figure S1, S2).pptx]

## Slide 1
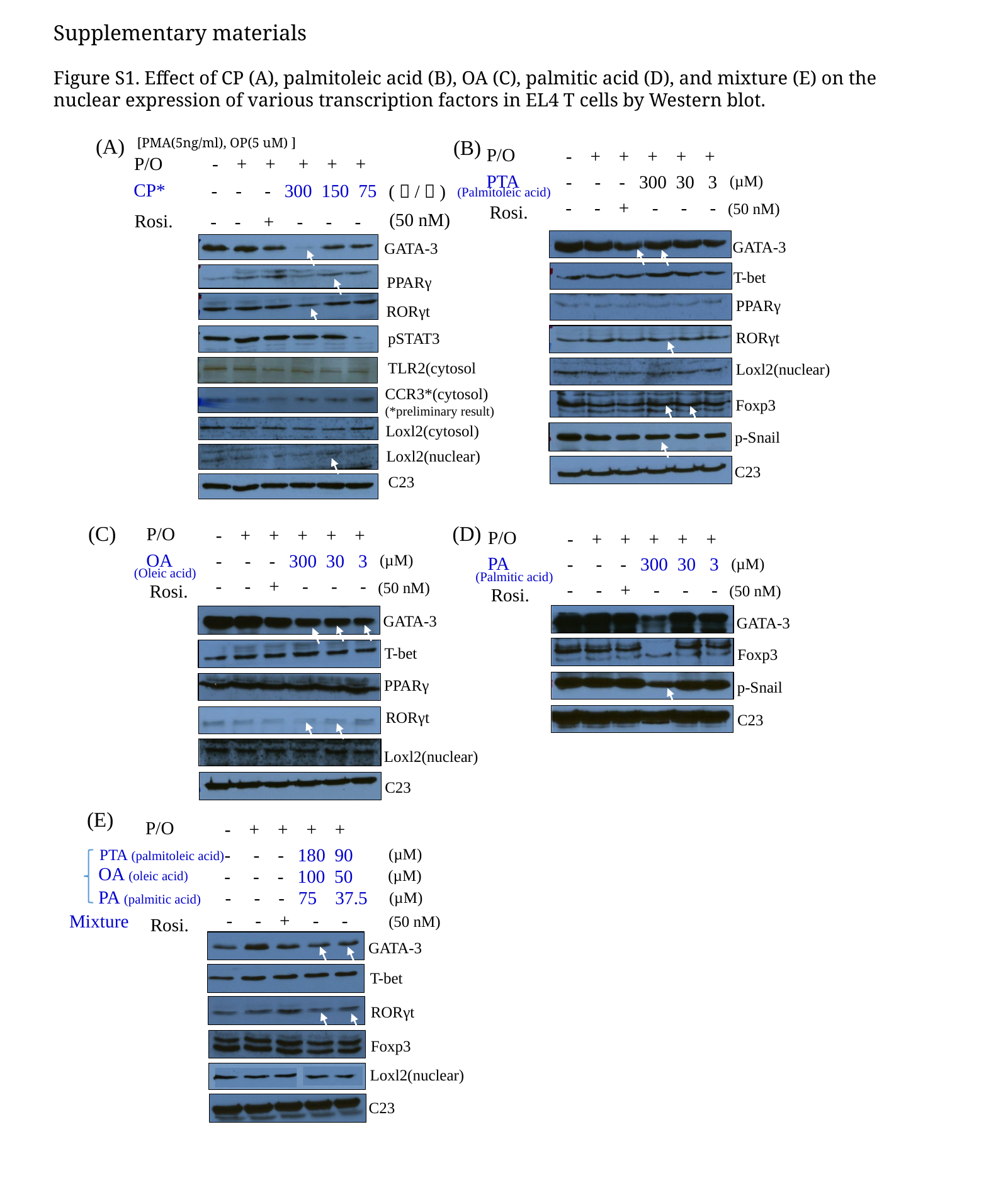

Supplementary materials
Figure S1. Effect of CP (A), palmitoleic acid (B), OA (C), palmitic acid (D), and mixture (E) on the nuclear expression of various transcription factors in EL4 T cells by Western blot.
(A)
(B)
[PMA(5ng/ml), OP(5 uM) ]
P/O
- + + + + +
P/O
- + + + + +
PTA
- - - 300 30 3
(µM)
CP*
- - - 300 150 75
(㎍/㎖)
(Palmitoleic acid)
- - + - - -
 (50 nM)
Rosi.
(50 nM)
Rosi.
- - + - - -
GATA-3
GATA-3
T-bet
PPARγ
PPARγ
RORγt
RORγt
pSTAT3
TLR2(cytosol
Loxl2(nuclear)
CCR3*(cytosol)
(*preliminary result)
Foxp3
Loxl2(cytosol)
p-Snail
Loxl2(nuclear)
C23
C23
(C)
(D)
P/O
- + + + + +
P/O
- + + + + +
OA
- - - 300 30 3
(µM)
PA
- - - 300 30 3
(µM)
(Oleic acid)
(Palmitic acid)
- - + - - -
 (50 nM)
- - + - - -
Rosi.
 (50 nM)
Rosi.
GATA-3
GATA-3
T-bet
Foxp3
PPARγ
p-Snail
RORγt
C23
Loxl2(nuclear)
C23
(E)
P/O
- + + + +
- - - 180 90
(µM)
PTA (palmitoleic acid)
OA (oleic acid)
- - - 100 50
(µM)
PA (palmitic acid)
- - - 75 37.5
(µM)
- - + - -
Mixture
 (50 nM)
Rosi.
GATA-3
T-bet
RORγt
Foxp3
Loxl2(nuclear)
C23

## Slide 2
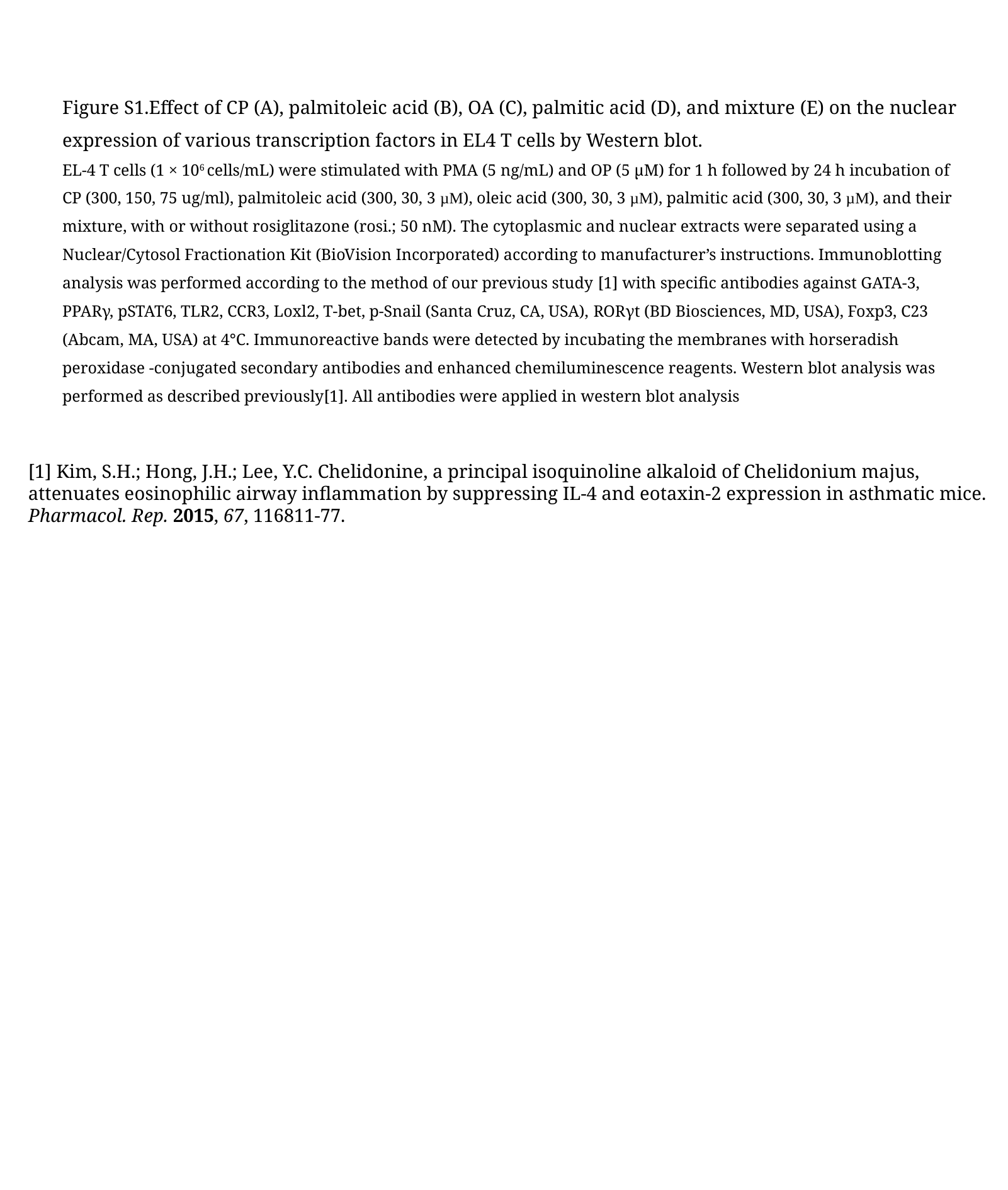

Figure S1.Effect of CP (A), palmitoleic acid (B), OA (C), palmitic acid (D), and mixture (E) on the nuclear expression of various transcription factors in EL4 T cells by Western blot.
EL-4 T cells (1 × 10^6^ cells/mL) were stimulated with PMA (5 ng/mL) and OP (5 µM) for 1 h followed by 24 h incubation of CP (300, 150, 75 ug/ml), palmitoleic acid (300, 30, 3 µM), oleic acid (300, 30, 3 µM), palmitic acid (300, 30, 3 µM), and their mixture, with or without rosiglitazone (rosi.; 50 nM). The cytoplasmic and nuclear extracts were separated using a Nuclear/Cytosol Fractionation Kit (BioVision Incorporated) according to manufacturer’s instructions. Immunoblotting analysis was performed according to the method of our previous study [1] with specific antibodies against GATA-3, PPARγ, pSTAT6, TLR2, CCR3, Loxl2, T-bet, p-Snail (Santa Cruz, CA, USA), RORγt (BD Biosciences, MD, USA), Foxp3, C23 (Abcam, MA, USA) at 4°C. Immunoreactive bands were detected by incubating the membranes with horseradish peroxidase -conjugated secondary antibodies and enhanced chemiluminescence reagents. Western blot analysis was performed as described previously[1]. All antibodies were applied in western blot analysis
[1] Kim, S.H.; Hong, J.H.; Lee, Y.C. Chelidonine, a principal isoquinoline alkaloid of Chelidonium majus,
attenuates eosinophilic airway inflammation by suppressing IL-4 and eotaxin-2 expression in asthmatic mice.
Pharmacol. Rep. 2015, 67, 116811-77.

## Slide 3
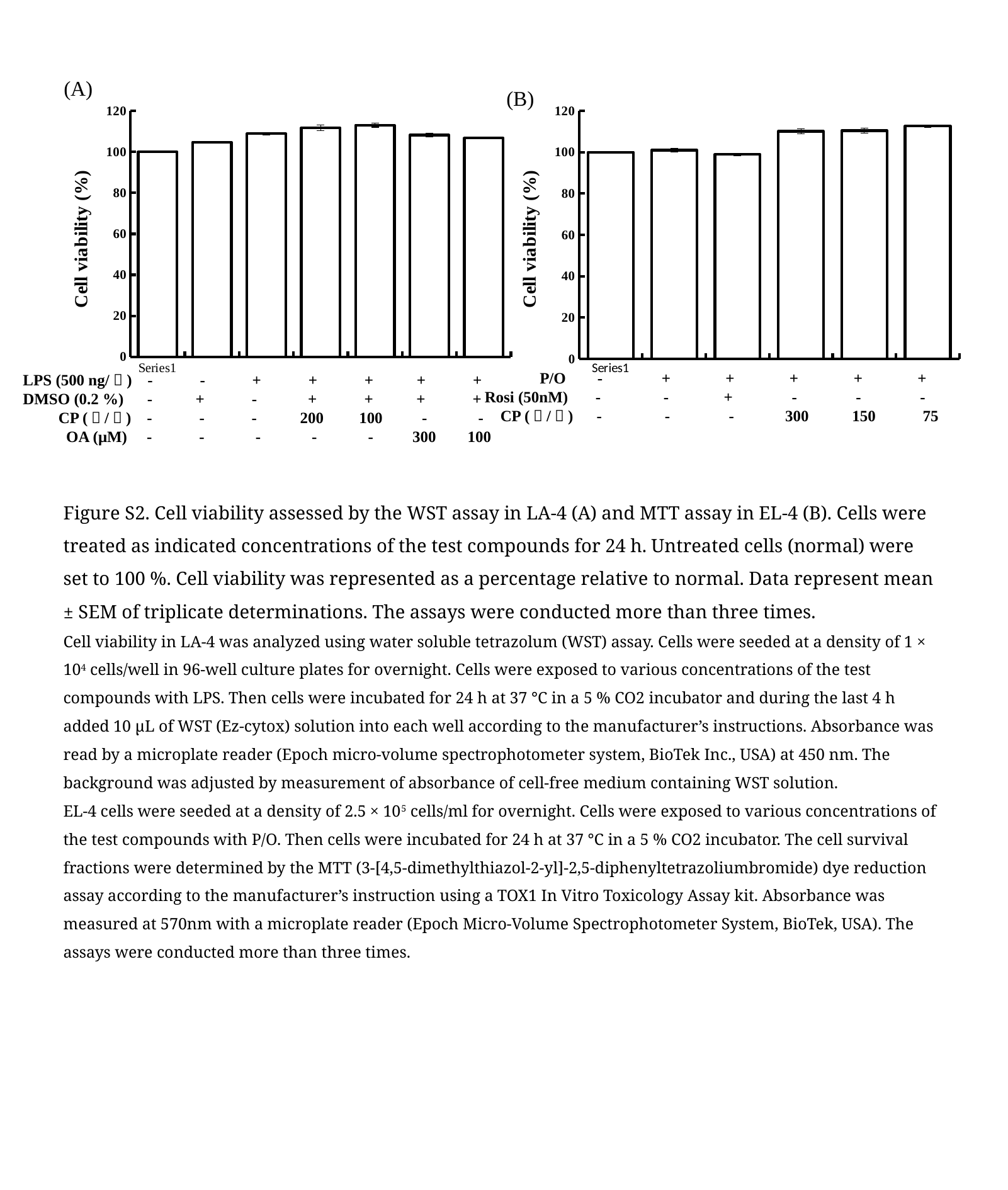

(A)
(B)
### Chart
| Category | |
|---|---|
| | 100.0 |
| | 104.71584154039755 |
| | 108.82721856448325 |
| | 111.72063115236153 |
| | 112.99169448074645 |
| | 108.17217247387573 |
| | 106.68724302434858 |
### Chart
| Category | |
|---|---|
| | 100.0 |
| | 100.96758496037499 |
| | 98.90066273395966 |
| | 110.08352036864164 |
| | 110.38277686191168 |
| | 112.59413007064308 | P/O - + + + + +
Rosi (50nM) - - + - - -
 CP (㎍/㎖) - - - 300 150 75
LPS (500 ng/㎖) - - + + + + +
DMSO (0.2 %) - + - + + + +
 CP (㎍/㎖) - - - 200 100 - -
 OA (μM) - - - - - 300 100
Figure S2. Cell viability assessed by the WST assay in LA-4 (A) and MTT assay in EL-4 (B). Cells were treated as indicated concentrations of the test compounds for 24 h. Untreated cells (normal) were set to 100 %. Cell viability was represented as a percentage relative to normal. Data represent mean ± SEM of triplicate determinations. The assays were conducted more than three times.
Cell viability in LA-4 was analyzed using water soluble tetrazolum (WST) assay. Cells were seeded at a density of 1 × 10^4^ cells/well in 96-well culture plates for overnight. Cells were exposed to various concentrations of the test compounds with LPS. Then cells were incubated for 24 h at 37 °C in a 5 % CO2 incubator and during the last 4 h added 10 μL of WST (Ez-cytox) solution into each well according to the manufacturer’s instructions. Absorbance was read by a microplate reader (Epoch micro-volume spectrophotometer system, BioTek Inc., USA) at 450 nm. The background was adjusted by measurement of absorbance of cell-free medium containing WST solution.
EL-4 cells were seeded at a density of 2.5 × 10^5^ cells/ml for overnight. Cells were exposed to various concentrations of the test compounds with P/O. Then cells were incubated for 24 h at 37 °C in a 5 % CO2 incubator. The cell survival fractions were determined by the MTT (3-[4,5-dimethylthiazol-2-yl]-2,5-diphenyltetrazoliumbromide) dye reduction assay according to the manufacturer’s instruction using a TOX1 In Vitro Toxicology Assay kit. Absorbance was measured at 570nm with a microplate reader (Epoch Micro-Volume Spectrophotometer System, BioTek, USA). The assays were conducted more than three times.
